# SWAP-Assembler: scalable and efficient genome assembly towards thousands of cores

**DOI:** 10.1186/1471-2105-15-S9-S2

**Published:** 2014-09-10

**Authors:** Jintao Meng, Bingqiang Wang, Yanjie Wei, Shengzhong Feng, Pavan Balaji

**Affiliations:** 1 Shenzhen Institutes of Advanced Technology, Chinese Academy of Sciences, 518055 Shenzhen, P.R. China; 2 Institute of Computing Technology, Chinese Academy of Sciences, 100190 Beijing, P.R. China; 3 University of Chinese Academy of Sciences, 100049 Beijing, P.R. China; 4 Beijing Genomics Institute, 518083 Shenzhen, P.R. China; 5 Mathematics and Computer Science Division, Argonne National Laboratory, 60439-4844 USA

**Keywords:** genome assembly, parallel computing, De Bruijn graph

## Abstract

**Background:**

There is a widening gap between the throughput of massive parallel sequencing machines and the ability to analyze these sequencing data. Traditional assembly methods requiring long execution time and large amount of memory on a single workstation limit their use on these massive data.

**Results:**

This paper presents a highly scalable assembler named as SWAP-Assembler for processing massive sequencing data using thousands of cores, where SWAP is an acronym for Small World Asynchronous Parallel model. In the paper, a mathematical description of multi-step bi-directed graph (MSG) is provided to resolve the computational interdependence on merging edges, and a highly scalable computational framework for SWAP is developed to automatically preform the parallel computation of all operations. Graph cleaning and contig extension are also included for generating contigs with high quality. Experimental results show that SWAP-Assembler scales up to 2048 cores on Yanhuang dataset using only 26 minutes, which is better than several other parallel assemblers, such as ABySS, Ray, and PASHA. Results also show that SWAP-Assembler can generate high quality contigs with good N50 size and low error rate, especially it generated the longest N50 contig sizes for Fish and Yanhuang datasets.

**Conclusions:**

In this paper, we presented a highly scalable and efficient genome assembly software, SWAP-Assembler. Compared with several other assemblers, it showed very good performance in terms of scalability and contig quality. This software is available at: https://sourceforge.net/projects/swapassembler

## Background

To cope with massive sequence data generated by next-generation sequencing machines, a highly scalable and efficient parallel solution for fundamental bioinformatic applications is important [1,2]. With the help of high performance computing, cloud computing [3,4], and many-cores in GPU [5], successful scalable examples have been seen in many embarrassingly parallel applications: sequence alignment [6-8], SNP searching [9,10], expression analysis [11], etc. However, for tightly coupled graph related problems, such as genome assembly, a scalable solution is a still a big challenge [12,13].

State-of-the-art trials on parallel assemblers include ABySS [14], Ray [15], PASHA [16], and YAGA [17-19]. ABySS adopts the traditional de Bruijn graph data structure proposed by Pevzner et. al. [20] and follows the similar assembly strategy as EULER SR [21] and Velvet [22]. The parallelization is achieved by distributing *k*-mers to multi-servers to build a distributed de Bruijn graph, and error removal and graph reduction are implemented over MPI communication primitives. Ray extends *k*-mers (or seeds) into contigs with a heuristical greedy strategy by measuring the overlapping level of reads in both direction. Based on the observation that the time consuming part of genome assembly are generating and distributing *k*-mers, constructing and simplifying the distributed de Bruijn graph, PASHA concentrates its effort on parallelizing these two stages to improve its efficiency. However, PASHA allows only single process for each unanimous path, and this limits its degree of parallelism. In their experiments, ABySS and PASHA take about 87 hours and 21 hours to assembly the Yoruban male genome with a coverage of 42X.

To avoid merging *k*-mers on two different servers, which can result in too many small inter-process messages and the communication latency, YAGA constructs a distributed de Bruijn graph by maintaining edge tuples in a community of servers. Reducible edges belonging to one unanimous path are grouped into one server using a list rank algorithm [23], then these unanimous paths are reduced locally on separated servers. The complexity of YAGA is bounded by $O\left(\frac{n}{p}\right)$ computing time, $O\left(\frac{n}{p}\right)$ communication volume, and *O*(*log*^2^(*n*)) communication rounds, where *n *is the number of nucleotides in all reads, and *p *denotes the number of processors. Due to the fact that the recursive list ranking algorithm used in YAGA has a memory usage of $O\left(\frac{n\text{log}n}{p}\right)$, this will use large amount of memory and cause low efficiency.

Our previous work [24] tries to avoid access collision of merging two neighbor edges. In this work, 1-step bi-directed graph and a computational model named as SWAP are proposed for edge merging operation. In its experiments, the prototype of edge merging algorithm using SWAP can scale to 640 cores on both Yeast and C.elegans dataset. However this exploratory work only focuses on the edge merging operation of genome assembly, some other important problems are not addressed, for example, contig extension, complexity analysis etc.

**Figure 1:**
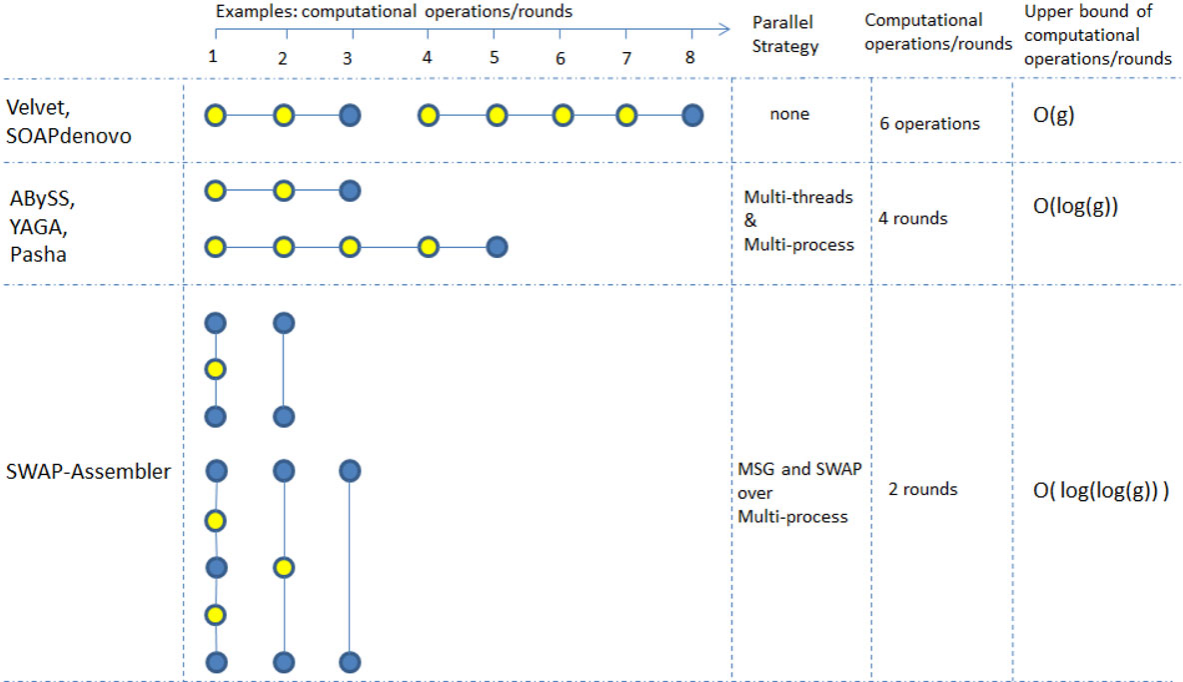
**Parallel strategy comparison on k-mers/edges merging for different assemblers**. Two linked paths with 3 nodes and 5 nodes are given as an example, merging a linked path with 3 nodes needs 2 operations/rounds, and merging a path with 5 nodes needs 4 operations/rounds. To assemble these two paths, sequential assemblers need 6 operations, and parallel assemblers need 4 rounds. For SWAP-Assembler different processes can merge several edges on the same path in parallel using the SWAP computational framework, and merging of these 2 paths can be finished in 2 rounds. For a given sequencing data, if we treat the sequencing coverage as an constant number, the upper bound of the three assemby strategies on merging k-mers/edges are bounded by O(g), O(log(g)), and O(log(log(g))) respecitively, here g denotes the genome size and the longest path for a genome of length g will be bounded by O(log(g)). The upper bound of edge merging operations in SWAP-Assembler and expected length of longest path are proved in Appedix 3. Reference of the k-mers merging strategy for these assemblers can be found in their papers or codes. For velvet 1.1.04, the k-mers merging method can be found in its code "./src/concatenatedGraph.c"; for SOAPdenovo-V1.05, its method is in the code "./src/31mer/contig.c"; for ABySS 1.3.5, the method can be found in "./Parallel/NetworkSequenceCollection.cpp"; for YAGA, the method has been descripted in the last paragraph in the methods section [18]; for Pasha, its method is presented in the last paragraph at the graph simplification subsection [16].

The scalability of previous assemblers is affected by the computational interdependence on merging *k*-mers/edges in unanimous paths. Sequential assemblers, for example Velvet and SOAPdenovo, process each path sequentially. Parallel assemblers can process several paths in parallel, however *k*-mers/edges sharing one path are merged one by one. SWAP-Assembler resolves the computational interdependence on merging edges sharing the same path with MSG. For each path, at most half of its edges can be merged concurrently in each round, and merging multiple edges on the same path can be done in parallel using SWAP computational framework. In Figure 1, the parallel strategy of SWAP-Assembler is compared with other assemblers using an example of two linked paths, we can see that a deeper parallelism on edge merging can be achieved by SWAP-Assembler.

In this paper, we present a highly scalable and efficient genome assembler named as SWAP-Assembler, which can scale to thousands of cores on processing massive sequencing data such as Yanhuang (500G). SWAP-Assembler includes five fully parallelized steps: input parallelization, graph construction, graph cleaning, graph reduction and contig extension. Compared with our previous work, two fundamental improvements have been made for graph reduction. Firstly MSG is presented as a comprehensive mathematical abstraction for genome assembly. Using MSG and semi-group theory, the computational interdependence on merging edges is resolved. Secondly, we have developed a scalable computational framework for SWAP, this framework triggers the parallel computation of all operations with no interference. In this paper, complexity of this framework and SWAP-Assembler is also analyzed and proved in detail. In addition, two steps in SWAP-Assembler are used to improve the quality of contigs. One is graph cleaning, which adopts the traditional strategy of removing *k*-molecules and edges with low frequency, and the other one is contig extension, which resolves special edges and some cross nodes using a heuristic method. Experimental results show that SWAP-Assembler can scale up to 2048 cores on Yanhuang dataset using only 26 minutes, which is the fastest compared with other assemblers, such as ABySS, Ray and PASHA. Conitg evaluation results confirm that SWAP-Assembler generates good results on N50 size with lowest error rate for *S. aureus *and *R. sphaeroides *datasets. When processing larger datasets (Fish and Yanhuang) without using external error correction tools, SWAP-Assembler generates the longest N50 contig sizes of 1309 bp and 1368 bp for these two datasets.

## Methods

In this section, our method for genome assembly towards thousands of cores is presented. We first abstract the genome assembly problem with MSG. Generating longer sequences (contigs) from shorter sequences corresponds to merging semi-extended edges to full-extended edges in MSG. In addition, computational interdependence of edge merging is resolved by introducing a semi-group over a closed edge set *Es *V **0 **in MSG. The edge set *Es *V **0 **is proved to be a semi-group with respect to edge merging operation. According to the associativity law of semi-group, the final results will be the same as long as all edges have been merged regardless of the merging order, thus these edge merging operations can be computed in parallel.

In order to maximally utilize the potential parallelism resolved by MSG, a scalable SWAP computational framework is developed. As one edge may be accessed by two merging operations in two different processes at the same time, a lock-computing-unlock mechanism introduced in [24] is adopted for avoiding the conflict. For the problems which can be abstracted with semi-group, the corresponding operations can be done in parallel, and SWAP computational framework can achieve linearly scale up for these problems.

Based on MSG and SWAP computational framework, SWAP-Assembler is developed with five steps, including input parallelization, graph construction, graph cleaning, graph reduction, and contig extension. In the following, we first present MSG and the SWAP computational framework, then details of SWAP-Assembler's five steps will be followed.

### Mathematical formulation of genome assembly using MSG

Given a biological genome sample with reference sequence *w ∈ *ℕ*^g ^*, where N = {*A, T, C, G*}, *g *= *|w|*, a large number of short sequences called **reads**, *S *= {*s*_1_*, s*_2_*, ..., s_h_*}, can be generated from the sequencing machines. Genome assembly is the process of reconstructing the reference genome sequence from these reads. Unfortunately, the genome assembly problem of finding the shortest string with all reads as its substring falls into a NP-hard problem [25].

**Figure 2:**
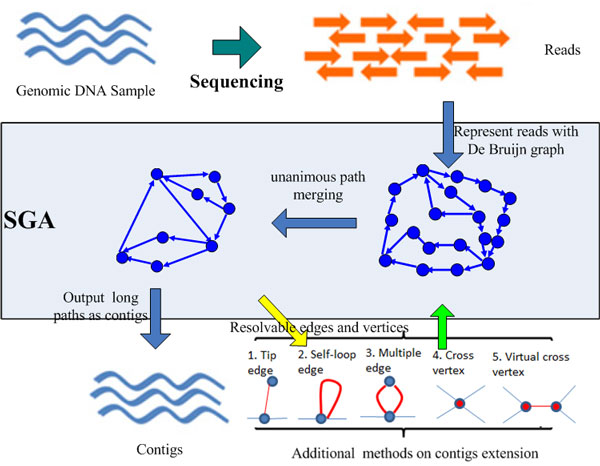
**The process of genome assembly and the standard genome assembly (SGA) problem**.

Finding the original sequence from all possible Euler paths cannot be solved in polynomial time [26]. In real cases, gaps and branches caused by uneven coverage, erroneous reads and repeats prevent obtaining full length genome, and a set of shorter genome sequences called **contigs **are generated by merging unanimous paths instead. Our method focuses on finding a mathematical and highly scalable solution for the following standard genome assembly (SGA) problem, which is also illustrated in Figure 2.

Problem of Standard Genome Assembly (SGA)

**Input**: Given a set of reads without errors *S *= {*s*_1_*, s*_2_*, ..., s_h_*}

**Output**: A set of contigs *C *= {*c*_1_*, c*_2_*, ..., c_w_*}

**Requirement**: Each contig maps to an unanimous path in the De Bruijn graph constructed from the set of reads *S*.

#### Preliminaries

We first define some variables. Let *s ∈ *ℕ*^l ^*be a DNA sequence of length *l*. Any substring derived from s with length *k*, is called a *k*-mer of *s*, and it is denoted by *α *= *s*[*j*]*s*[*j *+ 1] *. . . s*[*j *+ *k − *1], 0 ≤ *j < l − k *+ 1. The set of *k*-mers of a given string *s *can be written as Z(*s, k*), where *k *must be odd. The reverse complement of a *k*-mer *α*, denoted by α′, is obtained by reversing *α *and complementing each base by the following bijection of M, M : {*a *→ *t, t*→*a, c*→*g, g *→ *c*}. Note that $\alpha =\alpha^{\prime^{\prime }}$.

**Figure 3:**
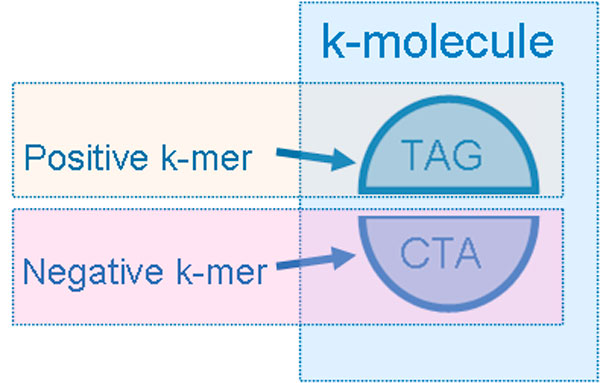
**Each k-molecule consists of one positive k-mer and one negative k-mer**.

A *k*-molecule $\hat{\alpha }$ is a pair of complementary *k*-mers {*α, α'*}. Let .> be the partial ordering relation between the strings of equal length, and α.>β indicates that *α *is lexicographically larger than *β*. We designate the lexicographically larger one of two complementary *k*-mers as the positive *k*-mer, denoted as *α*^+^, and the smaller one as the negative *k*-mer, denoted as *α−*, where $\alpha^{+}.>\alpha^{-}$. We choose the positive *k*-mer *α*^+ ^as representative *k*-mer for *k*-molecule {*α, α'*}, denoted as *α*^+^, implying that $\hat{\alpha }=\alpha^{+}=\left\{\alpha^{+},\alpha^{-}\right\}=\left\{\alpha ,\alpha \prime \right\}$. The relationship between *k*-mer and *k*-molecule is illustrated in Figure 3. The set of all *k*-molecules of a given string *s *is known as k-spectrum of *s*, and it can be written as S(*s, k*). Noted that S(*s, k*) = S(*s', k*).

Notation *suf *(*a, l*) (*pre*(*a, l*), respectively) is used to denote the length *l *suffix (prefix) of string *a*. The symbol ○ is introduced to denote the concatenation operation between two strings. For example, if *s*_1 _= "*abc*"*, s*_2 _= "*def *", then *s*_1 _°*s*_2 _= "*abcdef *". The number of edges attached to *k*-molecule *α*ˆ is denoted as *degree*($\hat{\alpha }$). All notations are listed in Table 1.

**Table 1:** List of notations.

Definition	Notation	Example
set of nucleotides	ℕ	ℕ = {*A, T, C, G*}
reference sequence	*W*	*w *= "*T AGT CGAGG*"
read set	*S*	*S *= { "*T AGT CG*", "*AGT CGA*", "*T CGAGG*" }
*k*-mer	*α *or *α^/^*	*α *= "*T AG*"*, α^/ ^*= "*CT A*"
positive *k*-mer	*_α_*+	*α*^+ ^= "*T AG*"
negative *k*-mer	*_α_−*	*α^− ^*= "*CT A*"
representative *k*-mer	*_α_*+	*α*^+ ^= "*T AG*"
*k*-molecule	^$\hat{\alpha }=\alpha^{+}=\left\{\alpha^{+},\alpha^{-}\right\}$^	$\hat{\alpha }$ = {"*T AG*", "*CT A*"}
set of *k*-mers	ℤ(*s, k*)	ℤ("*T AGT CG*", 3) ={"TAG","AGT","GTC","TCG"}
set of *k*-molecules	S(*s, k*)	ℤ("*T AGT C*", 3) = {{"*T AG*", "*CT A*"}, {"*AGT *", "*ACT *"}, {"*GT C*", "*GAC*"}, {"*T CG*", "*CGA*"}}

#### 1-step Bi-directed Graph

**Definition 1: 1-step bi-directed graph**. The 1-step bi-directed de Bruijn graph of order *k *for a given string *s *can be presented as,

(1) $$G_{k}^{1}\left(s\right)=\left\{Vs,E_{s}^{1}\right\}$$

In the rest of the paper, 1-step bi-directed de Bruijn graph of order *k *is abbreviated as 1**-step bi-directed graph**. In equation (1), the vertex set *V_s _*is the *k*-spectrum of *s*,

(2) $$V_{s}=S\left(s,k\right)$$

and the 1-step bi-directed edge set $E_{s}^{1}$ is defined as follows,

(3) $$\begin{array}{ccc} E_{s}^{1} & =\{e_{\alpha \beta }^{1}=\left(\alpha ,\beta ,d_{\alpha },d_{\beta },c_{\alpha \beta }^{1}\right)\forall \hat{\alpha },\hat{\beta }\in S\left(s,k\right),su\;f\left(\alpha ,k-1\right)= & \\ & \;\;pre\;\left(\beta ,k-1\right)ˆ\left(\alpha \circ \beta \left[k-1\right]\right)\in \left(Z\left(s,k+1\right)\vee Z\left(s\prime ,k+1\right)\right))\} \end{array}$$

Equations (3) declares that any two overlapped *k*-molecules can be connected with one 1-step bi-directed edge when they are consecutive in sequence *s *or the complementary sequence *s*'. Here *d_α _*denotes the direction of *k*-mer *α*, if *α *= *α*^+^, *d_α _*='+', otherwise *d_α _*= '-'. $c_{\alpha \beta }^{1}$ is the content or **label **of the edge, and is initialized with *β*[*k − *1], that is $c_{\alpha \beta }^{1}=\beta \left[k-1\right]$, and we have $su\;f\left(\alpha^{\circ }c_{\alpha \beta }^{1},k\right)=\beta$.

**Lemma 1**. Given two *k*-molecules $\hat{\alpha },\hat{\beta }\in S\left(s,k\right)$, there are four possible connections, and for each type of connection exactly two equivalent 1-step bi-directed edge representations exist,

1 $e_{\alpha +\beta +}^{1}=\left(\alpha^{+},\beta^{+},+,+,c_{\alpha +\beta +}^{1}\right),\;\;e_{\beta -\alpha -}^{1}=\left(\beta^{-},\alpha^{-},-,-,c_{\beta -\alpha -}^{1}\right)$

2 $e_{\alpha +\beta -}^{1}=\left(\alpha^{+},\beta^{-},+,-,c_{\alpha +\beta -}^{1}\right),\;\;e_{\beta +\alpha -}^{1}=\left(\beta^{+},\alpha^{-},+,=,c_{\beta +\alpha -}^{1}\right)$

3 $e_{\alpha -\beta +}^{1}=\left(\alpha^{-},\beta^{+},-,+,c_{\alpha -\beta -}^{1}\right),\;\;e_{\beta -\alpha +}^{1}=\left(\beta^{-},\alpha^{+},-,+,c_{\beta -\alpha +}^{1}\right)$

4 $e_{\alpha -\beta -}^{1}=\left(\alpha^{-},\beta^{-},-,-,c_{\alpha -\beta -}^{1}\right),\;\;e_{\beta +\alpha +}^{1}=\left(\beta^{+},\;\alpha^{+},+,+,c_{\beta +\alpha +}^{1}\right)$

**Figure 4:**
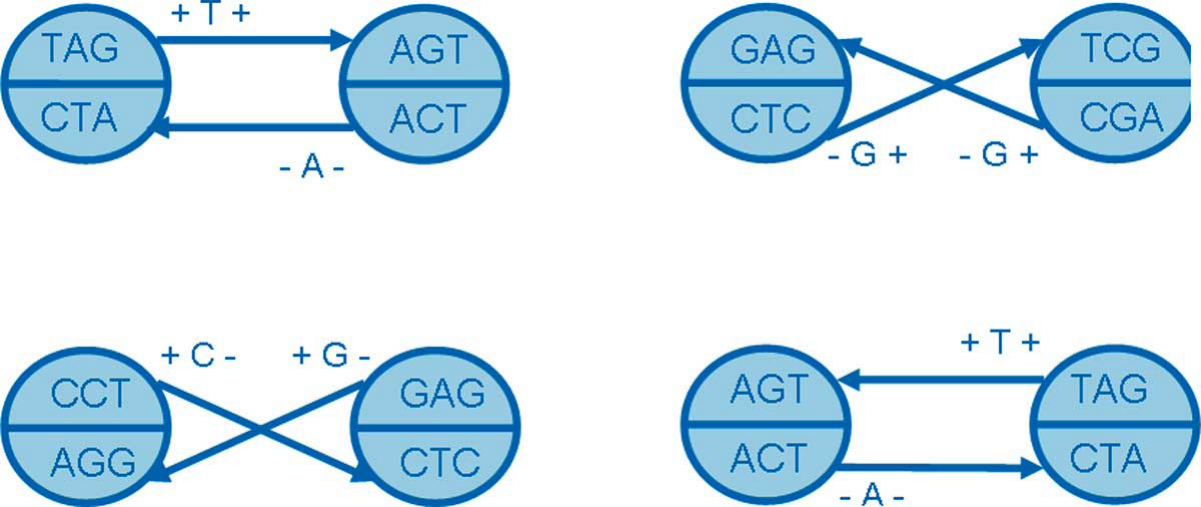
**The illustration of four possible connections**.

In each type of connection, the first bi-directed edge representation and the second one are equivalent. The first bi-directed edge is associated with *k*-molecule $\hat{\alpha }$, and the second one is associated with $\hat{\beta }$. Figure 4 illustrates all four possible connections. For example in Figure 4-(a), a positive *k*-mer "TAG" points to positive *k*-mer "AGT" with a label "A", and the corresponding edge is $e_{T\;AG\;AGT}^{1}=\left(TAG,AGT,+,+,T\right)$.

Given a set of reads *S *= {*s*_1_*, s*_2_*, . . . , s*_h_}, a 1-step bi-directed graph derived from *S *with order *k *is,

(4) $$G_{k}^{1}\left(S\right)=\left\{Vs,\;E_{S}^{1}\right\}=\left\{\underset{1\leq i\leq h}{\cup }V_{si},\underset{1\leq i\leq h}{\cup }E_{si}^{1}\right\}$$

**Figure 5:**
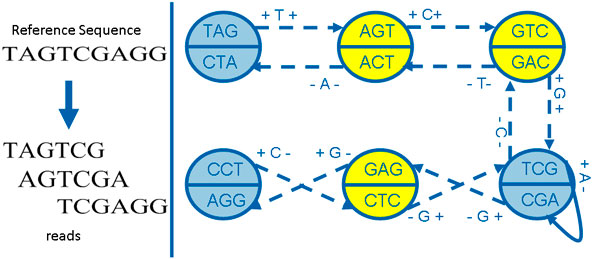
**An example of 1-step bi-directed graph**. Here semi-extended k-molecules are colored with yellow, and semi-extended edges are plotted with dashed line.

Each read *s_i _*corresponds to a path in $G_{k}^{1}\left(S\right)$, and read *s_i _*can be recovered by concatenating (*k *− 1)-prefix of the first *k*-molecule and the edge labels on the path consisted by S(*s_i_, k*). As an example, an 1-step bi-directed de Bruijn graph derived from *S *= {"*T AGT CG*", "*AGT CGA*", "*T CGAGG*"} is plotted in Figure 5.

#### Multi-step Bi-directed Graph and Its Properties

**Definition 2: edge merging operation**. Given two 1-step bi-directed edges $e_{\alpha \beta }^{1}=\left(\alpha ,\beta ,d_{\alpha },d_{\beta }.C_{\alpha \beta }^{1}\right)$ and $e_{\beta \gamma }^{1}=\left(\beta ,\gamma ,d_{\beta },d_{\gamma },C_{\beta \gamma }^{1}\right)$ in a 1-step bi-directed graph, if $e_{\alpha \beta }^{1}.d_{\beta }=e_{\beta \gamma }^{1}.d_{\beta }$ and *degree*($\hat{\beta }$) = 2, we can obtain a 2-step bi-directed edge $e_{\alpha \gamma }^{2}=\left(\alpha ,\gamma ,d_{\alpha },d_{\gamma },c_{\alpha \gamma }^{2}\right)$ by merging edges $e_{\alpha \beta }^{1}$ and $e_{\beta \gamma }^{1}$, $c_{\alpha \gamma }^{2}=c_{\alpha \beta }^{1}\circ c_{\beta \gamma }^{1}$. Using symbol ⊗ to denote **edge merging operation **between two bi-directed edges attached to the same *k*-molecule with the same direction, and the 2-step bi-directed edge is written as,

(5) $$e_{\alpha \beta }^{1}⊗e_{\beta \gamma }^{1}=e_{\alpha \gamma }^{2}\;\;\;or\;\;\;e_{\gamma \beta }^{1}⊗e_{\beta \alpha }^{1}=e_{\gamma \alpha }^{2}$$

**Figure 6:**
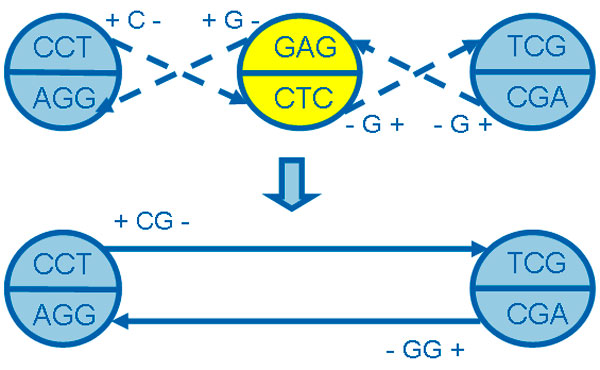
**An example for edge merging operation**.

Two edges $e_{\alpha \gamma }^{2}$ and $e_{\gamma \alpha }^{2}$ in equation (5) are equivalent, indicating it is same to apply edge merging operation on $e_{\alpha \beta }^{1}$ and $e_{\beta \gamma }^{1}$, and to apply edge merging operation on $e_{\gamma \beta }^{1}$ and $e_{\beta \alpha }^{1}$. Figure 6 shows an example on edge merging operation.

**Zero edge 0 **is defined to indicate all non-existing bi-directed edges. Note that $0⊗e_{\alpha \beta }^{x}=0,\;\;e_{\alpha \beta }^{x}⊗0=0$. A *z*-step bi-directed edge can be obtained by,

(6) $$e_{\alpha \gamma }^{z}=\left\{\begin{array}{cc} e_{\alpha \beta }^{x}⊗e_{\beta \gamma }^{y}, & if\exists \beta ,e_{\alpha \beta }^{x}\neq 0,\;e_{\beta \gamma }^{y}\neq 0,\;z=x+y\\ & e_{\alpha \beta }^{x}.d_{\beta }=e_{\beta \gamma }^{y}.d_{\beta },\;degree\left(\hat{\beta }\right)=2\\ 0 & \text{otherwise} \end{array}\right)$$

**Definition 3: Multi-step Bi-directed Graph(MSG)**. A MSG derived from a read set *S *= {*s*_1_*, s*_2_*, . . . , s_h_*}, is written as,

(7) $$G_{k}\left(S\right)=\left\{V_{S},E_{S}\right\}=\left\{\underset{1\leq i\leq h}{⋃}V_{si},\;\;\underset{1\leq j\leq g}{⋃}\left(\underset{1\leq i\leq h}{⋃}E_{si}^{j}\right)\right\}$$

where *g *is the length of reference sequence *w*, $E_{si}^{j}+\left\{e_{\alpha \beta }^{j}\text{|}\forall \hat{\alpha },\hat{\beta }\in S\left(s_{i},k\right)\right\}$. A MSG is obtained through edge merging operations.

Given an *x*-step bi-directed edge $e_{\alpha \beta }^{x}=\left(\alpha ,\beta ,d_{\alpha },d_{\beta },c_{\alpha \beta }^{x}\right)$, if there exists edge $e_{\gamma \alpha }^{y}$ or $e_{\beta \gamma }^{z}$ satisfying $e_{\gamma \alpha }^{y}⊗e_{\alpha \beta }^{x}\neq 0$ or $e_{\alpha \beta }^{x}⊗e_{\beta \gamma }^{z}\neq 0$, then we call edge $e_{\alpha \beta }^{x}$ as a **semi-extended edge**, and the corresponding *k*-molecule *α*ˆ or *β*ˆ as **semi-extended ***k***-molecule**. If $e_{\alpha \beta }^{x}$ cannot be extended by any edge, this edge is called as **full-extended edge**, and *k*-molecule $\hat{\alpha }$ and $\hat{\beta }$ are **full-extended ***k***-molecules**. In Figure 5 and 6, semi-extended *k*-molecule and full-extended *k*-molecule are plotted with different colors (yellow for semi-extended *k*-molecules and blue for full-extended *k*-molecules), semi-extended edge and full-extended edge are drawn with different lines (broken line for semi-extended edges and real line for full-extended edges).

**Property 1**. If the set of full-extended edges in the MSG defined in equation 7 is denoted as $E_{S}^{*}$, then the set of labels on all edges in $E_{S}^{*}$ can be written as,

(8) $$L_{S}^{*}=\left\{c_{\alpha \beta }^{x}\text{|}e_{\alpha \beta }^{x}=\left(\alpha ,\beta ,d_{\alpha },d_{\beta },c_{\alpha \beta }^{x}\right),\;e_{\alpha \beta }^{x}\in E_{S}^{*}\right\}$$

and we have $L_{S}^{*}=C$, C is the set of contigs. The proof is presented in Appendix 1.

**Property 2**. Edge merging operation ⊗ over the multi-step bi-directed edge set $E_{S}\vee 0$ is associative, and *Q*($E_{S}\vee 0$,⊗) is a semigroup. The proof is presented in Appendix 1.

The key property of 1-step bi-directed graph $G_{k}^{1}\left(S\right)$ is that each read *s *corresponds to a path starting from the first *k*-molecule of *s *and ending at the last *k*-molecule. Similarly, each chromosome can also be regarded as a path. However because of sequencing gaps, read errors, and repeats in the set of reads, chromosome will be broken into pieces, or contigs. Within a MSG, each contig corresponds to one full-extended edge in $G_{k}\left(S\right)$, and this has been presented and proved in Property 1. Property 2 ensures that the edge merging operation ⊗ over the set of multi-step bi-directed edges has formed a semi-group, and this connects the standard genome assembly (SGA) problem with edge merging operations in semi-group. According to the associativity law of semi-group, the final full-extended edges or contigs will be the same as long as all edges have been merged regardless of the merging order, thus these edge merging operations can be computed in parallel. Finally in order to reconstruct the genome with a large set of contigs, we need to merge all semi-extended edges into full-extended edges in semi-group *Q*($E_{S}\vee 0$, ⊗).

### SWAP computational framework

The lock-computing-unlock mechanism of SWAP was first introduced in our previous work [24], where no implementation details and complexity analysis is given. In this section, we present the mathematical description of the problems which can be solved by SWAP, then a scalable computational framework for SWAP model and its programming interface are presented. Its complexity and scalability is analyzed in Appendix 3.

**Definition 4: small world of operations**. Semi-group *SG*(*A, R*) is defined on set *A *with an associative operation *R *: *A × A *→ *A. R*(*a_i_, a_j_*) is used to denote the associative operation on *a_i _*and *a_j_, a_i_, a_j _*∈ *A*. The elements *a_i _*and *a_j_*, together with the operation *R*(*a_i_, a_j _*) are grouped as a **small world **of the operation R(*a_i_, a_j_*). We denote this small world as [*a_i_, a_j_*], and [*a_i_, a_j_*] = {*R*(*a_i_, a_j_*)*, a_i_, a_j_*}.

**Activity ***ACT *(*A*, σ) are given on a semi-group *SG*(*A, R*) as the computational works performed by a graph algorithm, where operation set *σ *is a subset of *R*.

In real application, an operation corresponds to a basic operation of a given algorithm. For example, for MSG based genome assembly application, an operation can be defined as edge merging. For topological sorting, re-ordering a pair of vertices can be defined as an operation.

For any two small worlds [*a*_1_*, a*_2_], [*b*_1_*, b*_2_], where *a*_1 _≠ *b*_1_*, a*_1 _*I*= *b*_2_*, a*_2 _≠ *b*_1_*, a*_2 _≠ *b*_2_, the corresponding operations *R*(*a*_1_*, a*_2_) and *R*(*b*_1_*, b*_2_), can be computed independently, thus, there exists potential parallelism in computing activity induced from the semi-group *SG*(*A, R*). We use SWAP for such parallel computing. The basic schedule of SWAP is **Lock**-**Computing**-**Unlock**. For an operation *R*(*a, b*) in *σ*, the three-steps of SWAP are listed below:

1 **Lock **action is applied to lock *R*(*a, b*)'s small world [*a, b*].

2 **Computing **is performed for operation *R*(*a, b*), and the values of *a, b *are updated accordingly. In MSG, this corresponds to merging two edges.

3 **Unlock **action is triggered to release operation *R*(*a, b*)*!s *small world [*a, b*].

In SWAP computational framework, all operations *σ *in activity *ACT *(*A, σ*) can be distributed among a group of processes. Each process needs to fetch related elements, for example *a *and *b*, to compute operation *R*(*a, b*). At the same time, this process also has to cooperate with other processes for sending or updating local variables. Each process should have two threads, one is SWAP thread, which performs computing tasks using the three-steps schedule of SWAP, and the other is service thread, which listens and replies to remote processes. In the implementation of our framework, we avoid multi-threads technology by using nonblocking communication and finite-state machine in each process to ensure its efficiency.

**Figure 7:**
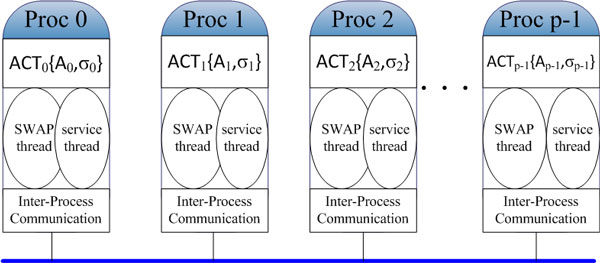
**Illustration of the distribution of an activity ACT (A, σ) on a cluster using SWAP computational framework**. Operation set σ is distributed over p processors, here $\sigma =\overset{p-1}{\underset{i=0}{⋃}}\sigma_{i}\overset{p-1}{\underset{i=0}{⋂}}\sigma_{i}=0$ and $ACT\left(A,\sigma \right)=\overset{p-1}{\underset{i=0}{⋃}}ACT_{i}\left(A_{i},\sigma_{i}\right)$.

The activity *ACT *(*A, σ*) on set *A *with operations in *σ *can be treated as a graph *G*(*σ, A*) with *σ *as its vertices and *A *as its edges. Adjacent list is used to store the graph *G*(*σ, A*), and a hash function *hashF un*(*x*) is used to distribute the set *σ *into *p *subset for *p *processes, where $\sigma =\overset{p-1}{\underset{i=0}{⋃}}\sigma_{i}$, and *A_i _*is associated with *σ_i_*. Note that $ACT\left(A,\sigma \right)=\overset{p-1}{\underset{i=0}{⋃}}ACT_{i}\left(A_{i},\sigma_{i}\right)$, which is illustrated in Figure 7. In Appendix 2, the pseudocodes of SWAP thread and service thread are demonstrated in Algorithm 1 and Algorithm 2, respectively.

Algorithm 1 describes the three-steps of SWAP computational framework, and Algorithm 2 on remote processers can cooperate with Algorithm 1 for running this schedule. The message functions, internal functions, and user-defined functions in Algorithm 1 and Algorithm 2 are listed in Table 2, where user-defined functions can be redefined for user-specific computational problems.

**Table 2:** Description of message functions, internal functions, and user-defined functions used in Algorithm 1 and 2.

Class	Function name	Function description
Message functions	Msg Lock(*a, p*)	Lock *a *in process *p*
	Msg Unlock(*a, p*)	Unlock *a *in process *p*
	Msg Read(*a, p*)	Fetch associated values of *a*
	Msg Write(*a, newa, p*)	Update associated values of *a *with *newa*
	Msg Locksuccess(*a, R*(*a, b*), *p*)	Send Locksuccess Message back to *R*(*a, b*)
	Msg Lockfailed(*a, R*(*a, b*), *p*)	Send Lockfailed Message back to *R*(*a, b*)
	Msg ReadBack(*a, R*(*a, b*), *p*)	Send associated value of *a *back to *R*(*a, b*)
	Msg End()	Command to stop the service thread
Internal functions	proc(*a*)	Get process ID of *a*
	trylock(*a*)	Lock a
	unlock(*a*)	Unlock *a*
User-defined functions	GetSmallWorld( *R*(*a, b*) )	Get small world [*a, b*] from operation *R*(*a, b*)
	Operation(*a,b*)	Compute the operation *R*(*a, b*)

Similar to CSMA/CA in 802.11 protocol [27], Algorithm 1 adapts random back-off algorithm to avoid lock collision. A variety of backoff algorithms can be used, without loss of generality, binary exponential backoff [27] is used in SWAP thread. Note that all collided operations in *σ_i _*share only one binary backoff, so the cost can be ignored as long as the number of relations in *σ_i _*is huge.

The complexity and scalability analysis for SWAP computational framework are presented in Appendix 3. When the number of processes is less than the number of operations in *σ*, which is true for most cases, equation (**??**) shows that SWAP computational framework can linearly scale up with the increasing number of cores. When the number of processes is larger than the number of operations, according to equation (**??**) the running time will be dominated by the communication round, which is bounded by *log*(*d_max_*), where *d_max _*is the diameter of graph *G*(*σ, A*).

### Implementation of SWAP-Assembler

Based on MSG and SWAP computational framework, SWAP-Assembler consists with five steps, including input parallelization, graph construction, graph cleaning, graph reduction, and contig extension. Complexity analysis of SWAP-Assembler are presented in the end of this section.

#### Input parallelization

As the size of data generated by next generation sequencing technology generally has hundreds of Giga bytes, loading these data with one process costs hours to finish [14,16]. Similar to Ray [15] and YAGA [18], we use input parallelization to speedup the loading process. Given input reads with *n *nucleotides from a genome of size *g*, we divide the input file equally into *p *virtual data block, *p *is the number of processes. Each process reads the data located in its virtual data block only once. The computational complexity of this step is bounded by $O\left(\frac{n}{p}\right)$. For E.coli dataset of 4.4G bytes, SWAP-Assembler loads the data into memory in 4 seconds with 64 cores while YAGA uses 516.5 seconds [18], and for Yanhuang dataset SWAP-Assembler loads the data in 10 minutes while Ray costs 2 hour and 42 minutes.

#### Graph construction

This step aims to construct a 1-step bi-directed graph $G_{k}^{1}\left(S\right)=\left\{V_{s},E_{s}^{1}\right\}$, where *V_s _*and $E_{s}^{1}$ are *k*-molecule set and 1-step bi-directed edge set. In this step, input sequences are broken into overlapping *k*-molecules by sliding a window of length *k *along the input sequence. A *k*-molecule can have up to eight edges, and each edge corresponds to a possible one-base extension, {*A, C, G, T*} in either direction. The adjacent *k*-molecule can be easily obtained by adding the base extension to the source *k*-molecule. The generated graph has *O*(*n*) *k*-molecules and *O*(*n*) bi-directed edges distributed among *p *processors. Graph construction of 1-step bi-directed graph can be achieved in $O\left(\frac{n}{p}\right)$ parallel computing time, and $O\left(\frac{n}{p}\right)$ parallel communication volume.

An improvement to our previous work [24] is that the time usage on graph construction is overlapped with the previous step. As CPU computation and network communication can be performed when only partial data are loaded from the first step, they can be overlapped and combined as a pipeline. Computation and communication time used in this step are hid behind the time used on disk I/O in previous step.

#### Graph cleaning

This step cleans the erroneous *k*-molecules, based on the assumption that the erroneous *k*-mers have lower frequency compared with the correct ones [19]. Assuming that the errors are random, we identify the *k*-molecules with low frequency as erroneous *k*-molecules, and delete them from the vertex set. SWAP-Assembler also removes all edges with low frequency in the 1-step bi-directed graph, and the *k*-molecules without any attached edges. The frequency threshold can be set by users, or our method will calculate it automatically based on the average coverage of *k*-molecules. In our case, we prefer 3 ~ 10% of the average coverage as the threshold depending on the species.

All the operations in this step can be finished in $O\left(\frac{n}{p}\right)$ parallel computation time, and about 60 ~ 80% of the *k*-molecules can be removed from our graph.

#### Graph reduction

In order to recover contigs, all semi-extended edges in MSG need to be merged into full-extended edges. This task can be defined as edge merging computing activity and denoted as *ACT *(Es∨0,σ), where the edge merging operation set *σ *is,

(9) $$\sigma =\left\{\left(e_{\beta \alpha }^{u},e_{\alpha \gamma }^{v}\right){|e}_{\beta \alpha }^{v}⊗e_{\alpha \gamma }^{v}\neq 0,e_{\beta \alpha }^{u},e_{\alpha \gamma }^{v}\in E_{s}\right\}$$

in which $e_{\beta \alpha }^{u}$ indicates an *u*-step bi-directed edge connecting two vertices *β *and *α*. All semi-extended edges of *E_s _*will be merged into full-extended edges finally.

In order to compute edge merging operations in *σ_i _*using our SWAP computational framework, two user-defined functions in Table 2 are described as Algorithm 3 and Algorithm 4 in Appendix 2. For each process, the edge merging step has a computing complexity of $O\left(\frac{n}{p}\right)$, communication volume of $\left(\frac{n\;\text{log}\left(\text{log}\left(g\right)\right)}{p}\right)$, and communication round of *O*(log(log(*g*))). The proposed methods has much less computation round of *O*(*loglog*(*g*)) than YAGA with *O*(*log*(*n*)2) [18,19]. The detailed complexity analysis is provided in Appendix 4.

#### Contig extension

In order to extend the length of contigs while maintaining as less errors as possible, three types of special edges and two type of special vertices are processed in our method.

The first type of special edge is tip edge, which is connected with an terminal vertex and has a length less than *k*, where *k *is the *k*-mer size. These tip edges are deleted from the graph. The second type is self-loop edge, whose beginning vertex and terminal vertex are same. If this vertex has another edge which can be merged with this self-loop edge, they will be merged, otherwise it will be removed. The last type is multiple edge, whose two vertices are directly connected by two different edges. In this case the edge with lower coverage will be removed.

**Figure 8:**
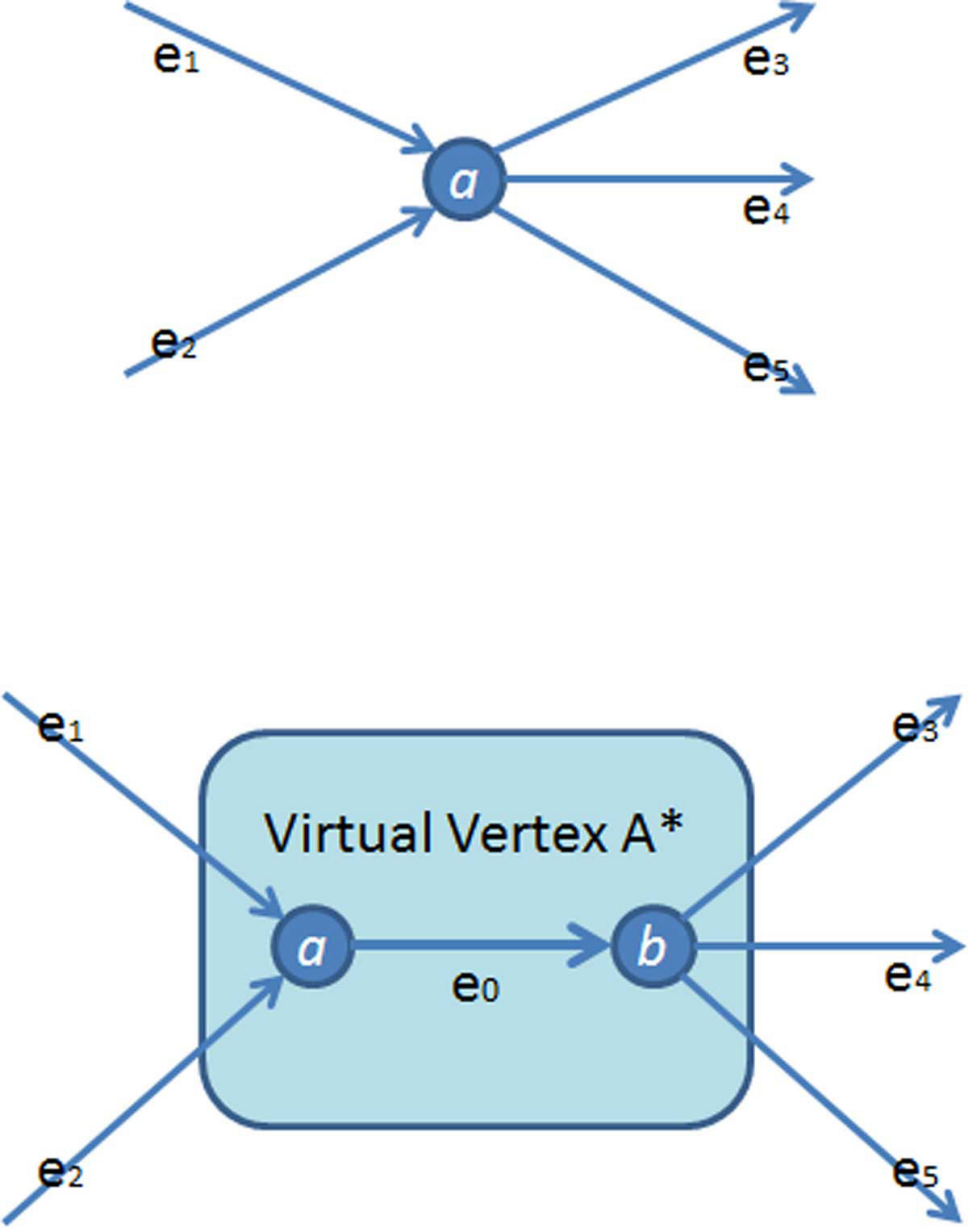
**Two type of special vertex defined in contig extension step**.

In addition, processing two special vertices can help further improve the quality of contigs. The first is cross vertex shown in Figure 8-(e), which has more than two edges on both sides. For each cross vertex, we sort all its edges according to their coverage. When the coverage difference between the two edges is less than 20%, then these two edges are merged as long as they can be merged regardless of other edges. The second vertex is virtual cross vertex shown in Figure 8-(f). We treat edge *e*_0 _with its two end vertices as one virtual vertex A* and A* has more than two edges on both sides. All its edges are ranked according to their coverage. When the coverage difference between two edges on different nodes is less than 20%, these two edges will be merged with the edge *e*_0 _regardless of other edges. By processing these two special vertices using the heuristic method, we can partly resolve some of the repeats satisfying our strict conditions at the cost of introducing errors and mismatches into contigs occasionally.

The graph reduction step and contig extension step need to be iterated in a constant number of rounds to extend full-extended edges or stop when no special edges and special vertices can be found. The number of errors and mismatches introduced in contigs can be controlled by the percentage of special edges and vertices processed in contig extension stage. In our method, we process all edges and vertices aiming at obtaining longer contigs. The computing complexity for contig extension step will be bounded by $O\left(\frac{n}{p}\right)$. As the graph shrinks greatly after graph reduction and contig extension step, all the remaining edges are treated as contigs. The complexity of SWAP-Assembler is dominated by graph reduction step, which is bounded by $O\left(\frac{n}{p}\right)$ parallel computing time, $O\left(\frac{n\cdot \text{log}\left(\text{log}\left(g\right)\right)}{p}\right)$ communication volume, and *O*(log(log(*n*))) communication round. According to complexity analysis results of graph reduction step in equation (**??**), when the number of processors is less than the length of longest path *d_max_*, the speedup of SWAP-Assembler can be calculated as follows,

(10) $$Speedup=\frac{n}{Run\;Time}=\frac{p}{bL\;\text{log}\left(3cq\cdot \text{log}\left(g\right)\right)+rS+1}$$

Equation (10) indicates that, for a given genome with fixed length *g*, the speedup is proportional to the number of processors; while for a given number of processors *p*, the speedup is inversely proportional to logarithm of the logarithm of the genome size *g*. However when the number of processors is larger than the length of longest path *d_max_*, the running time will be bounded by the number of communication round, which is presented in equation (**??**) in Appendix 4. In either situation, we can conclude that the scalability or the optimal number of cores will increase with larger genomes.

## Results

SWAP-Assembler is a highly scalable and efficient genome assembler using multi-step bi-directed graph (MSG). In this section, we perform several computational experiments to evaluate the scalability and assembly quality of SWAP-Assembler. In the experiments, TianHe 1A [28] is used as the high performance cluster. 512 computing nodes are allocated for the experiment with 12 cores and 24GB memory on each node. By comparing with several state-of-the-art sequential and parallel assemblers, such as Velvet [22], SOAPdenovo [29], Pasha [16], ABySS [14] and Ray [15], we evaluate the scalability, quality of contigs in terms of N50, error rate and coverage for SWAP-Assembler.

### Experimental data

Five datasets in Table 3 are selected for the experiments. *S. aureus, R. sphaeroides *and *human chromosome 14 *(Hg14) datasets are taken from GAGE project [30], Fish dataset is downloaded from the Assemblathon 2 [31,32], and Yanhuang dataset [33] is provided by BGI [34].

**Table 3:** Details about the five short read datasets.

-	*S. aureus*	*R. sphaeroides*	Hg14	Fish	YanHuang
Fastq data size (Gbytes)	0.684	0.906	14.2	425	495
Read length (bp)	37, 101	101	101	101	80-120
No. of reads (million)	4.8	4.1	62	1910	1859
Coverage	90X	90X	70X	192X	57X
Reference size (Mbp)	2.90	4.60	88.6	1000	3000

### Scalability evaluation

**Figure 9:**
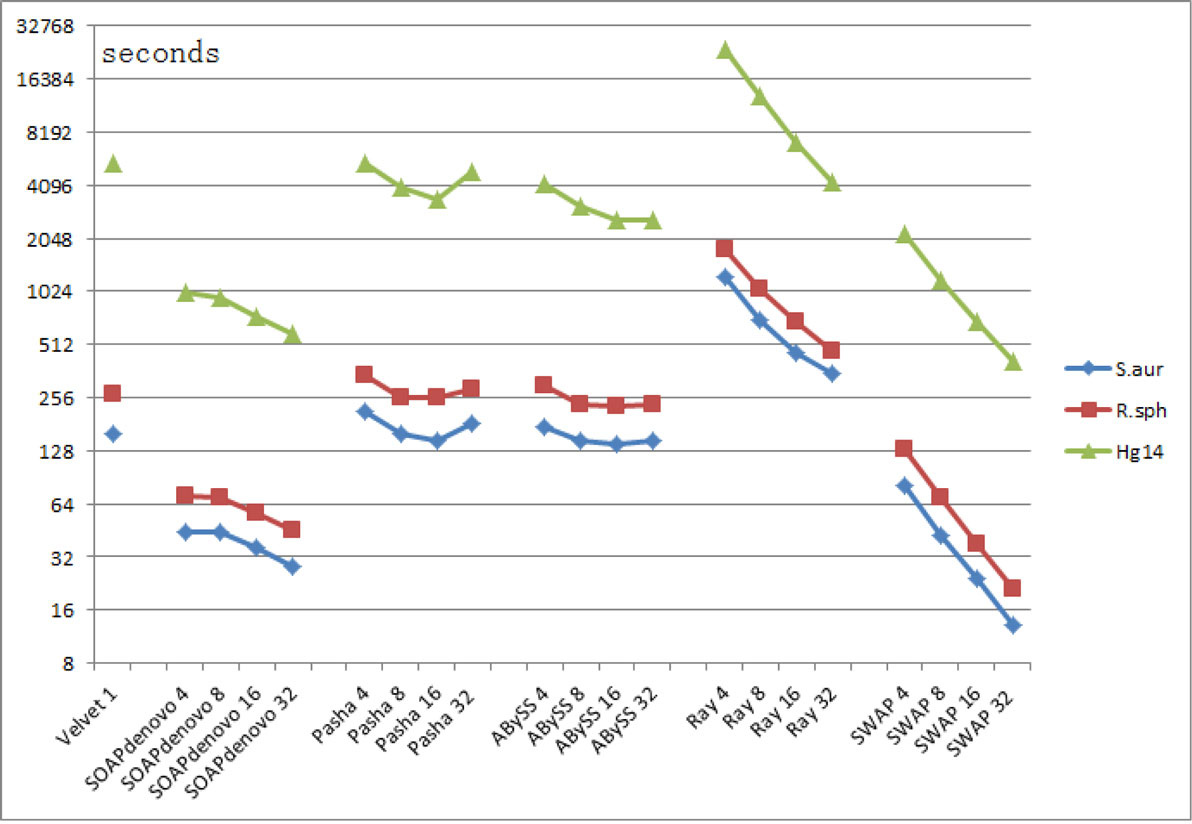
**Time usage comparison on a share memory machine for three small datasets (Time unit: seconds in logarithmic scale)**. In this test, the length of k-mer for all assemblers is set to be 31 and the k-mers cutoff threshold is set to be 3. For the three datasets, the sequencing data filtered by ALLPATH-LG is their input data. The time usage is recorded until the contig is generated. The horizontal axis is marked with the name of assemblers and the number of cores used.

The scalability of our method is first evaluated on a share memory machine with 32 cores and 1T memory. Five other assemblers including Velvet, SOAPdenovo, Pasha, ABySS and Ray, are included for comparison. Only the first three small datasets in Table 3 are used in this test due to the memory limitation. The results are presented in Table 4, and the corresponding figures are plotted in Figure 9. According to Table 4, SWAP-Assembler has the lowest running time on all three datasets for 16 cores and 32 cores, and SOAPdenovo has the lowest time usage on 4 cores and 8 cores. According to Figure 9, SOAPdenovo, Ray and SWAP-Assembler can scale to 32 cores, however Pasha and ABySS can only scale to 16 cores. Figure 9 also shows that Ray and SWAP-Assembler can achieve nearly linear speedup and SWAP-Assembler is more efficient than Ray.

**Table 4:** Time usage results of three small datasets on a share memory machine with 32 cores.

Assembler	Cores	*S. aureus*	*R. sphaeroides*	Hg14
Velvet	1	159	265	5432
SOAPdenovo	4	44	71	1004
	8	44	69	933
	16	36	57	742
	32	38	45	584
Pasha	4	215	342	5494
	8	159	255	3938
	16	147	255	3436
	32	183	289	4852
ABySS	4	174	302	4138
	8	146	234	3079
	16	139	226	2588
	32	147	235	2596
Ray	4	1247	1778	24145
	8	707	1050	13116
	16	454	688	7222
	32	351	467	4235
SWAP-Assembler	4	81	129	2167
	8	42	69	1187
	16	24	38	685
	32	13	21	408

(Time unit: seconds)

**Figure 10:**
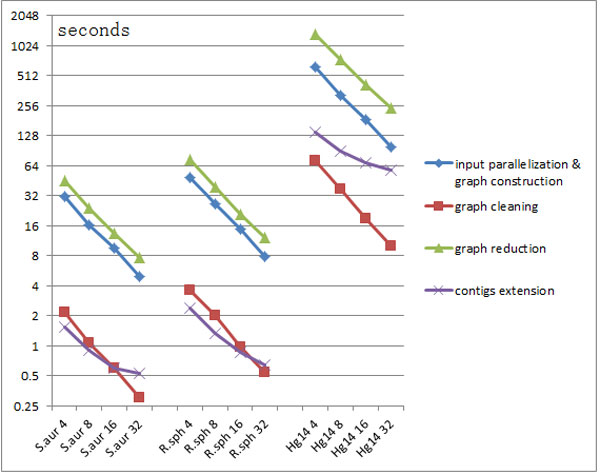
**Time usage details of SWAP-Assembler's five steps on processing three small datasets using a share memory machine with 32 cores (Time unit: seconds in logarithmic scale)**. In this test, the length of k-mer for SWAP-Assembler is set to be 31 and the k-mers cutoff threshold is set to be 3. For the three datasets, the sequencing data filtered by ALLPATH-LG is their input data. The horizontal axis is marked with the name of datasets and the number of cores used.

The time usage for each step of SWAP-Assembler on the share memory machine is also presented in Table 5 and Figure 10. The input parallelization step is overlapped with graph construction, thus we treat these two steps as one in this experiment. According to Table 5, for all three datasets the most time-consuming step is graph reduction, and the fastest steps are graph cleaning and contig extension. Figure 10 shows that input parallelization & graph construction, graph cleaning and graph reduction can achieve nearly linear speedup when the number of cores increases from 4 to 32 cores, whereas the contig extension step does not benefit as much as other steps.

**Table 5:** Time usage details of SWAP-Assembler's five steps on processing three small datasets using a share memory machine with 32 cores.

	Steps	4 cores	8 cores	16 cores	32 cores
*S. aureus*	input parallelization & graph construction	31.29	16.32	9.55	4.91
	graph cleaning	2.14	1.07	0.6	0.3
	graph reduction	45.75	24.2	13.23	7.67
	contig extension	1.54	0.89	0.6	0.52
*R. sphaeroides*	input parallelization & graph construction	49.19	26.42	14.88	7.79
	graph cleaning	3.63	1.99	0.97	0.54
	graph reduction	73.78	39.31	20.83	12.19
	contig extension	2.37	1.33	0.85	0.64
Hg14	input parallelization & graph construction	628	327	184	98
	graph cleaning	71	37	19	10
	graph reduction	1328	734	413	242
	contig extension	140	89	69	58

(Time unit: seconds)

To further evaluate the scalability of SWAP-Assembler from 64 to 4096 cores on TianHe 1A, we have compared our method with two parallel assemblers, ABySS and Ray, and the results are included in Table 6. According to Table 6 SWAP-Assembler is 119 times and 73 times faster than ABySS and Ray for 1024 cores on the *S. aureus *dataset. On the same dataset, ABySS and Ray cannot gain any speedup beyond 64 and 128 cores, respectively. However SWAP-Assembler scales up to 512 cores. For the *R. sphaeroides *dataset, ABySS, Ray, and SWAP-Assembler can scale up to 128, 256, and 1024 cores, respectively.

**Table 6:** Scalability evaluation on parallel assemblers 1 (Time unit: seconds).

dataset	software	64	128	256	512	1024	2048	4096
*S. aureus *(2.87 Mb)	ABySS	248	269	334	554	831	_-2_	-
	Ray	244	198	202	266	510	-	-
	SWAP-Assembler	23	15	8	5	7	13	-
*R. sphaeroides *(4.60 Mb)	ABySS	492	454	522	718	1312	-	-
	Ray	287	183	181	190	285	-	-
	SWAP-Assembler	43	29	15	7	7	7	-
Hg14 (88.29 Mb)	ABySS	6472	5299	6935	9045	16530	-	-
	Ray	2926	1746	1288	1517	2266	-	-
	SWAP-Assembler	585	428	203	128	59	67	-
Fish (1 Gb	ABySS	_* _3	_4_	+	+	+	-	-
	Ray	*	+	+	+	+	-	-
	SWAP-Assembler	+	13941	8622	3263	962	971	2582
Yanhuang (3 Gb)	ABySS	*	+	+	+	+	-	-
	Ray	*	+	+	+	+	-	-
	SWAP-Assembler	*	11243	5761	4021	1783	1608	1778

1 This table records the time usage on assembling all five datasets with different number of cores, and the length of *k*-mer is set to be 23. For ABySS and Ray, the time is recorded until contigs are generated.

2 − denotes assembler with this parameter has not been tested.

*3 ∗ *denotes assembler with this parameter has run out of memory.

4 + denotes assembler with this parameter has run out of time, the time limit is 12 hours.

**Figure 11:**
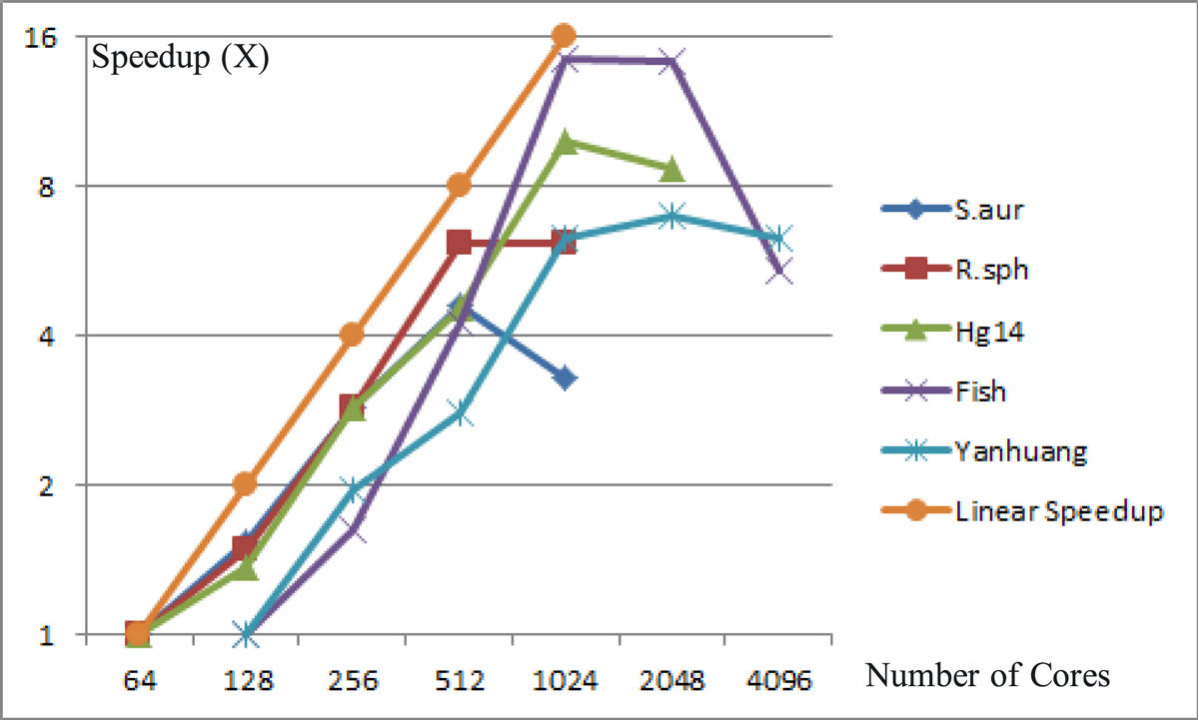
**Linear speedup of SWAP-Assembler on processing five datasets**.

For three larger datasets, Table 6 shows that scalability of SWAP-Assembler is also better than the other two methods. On Hg14 dataset, SWAP-Assembler is 280 times faster than ABySS, and 38 times faster than Ray when using 1024 cores. Similar to the results on *R. sphaeroides *dataset, three assemblers still hold their turning point of scalability at 128, 256 and 1024 cores, respectively. Fish and Yanhuang dataset cannot be assembled by ABySS and Ray in 12 hours, so their running times are not recorded in Table 6. For 1024 cores, SWAP-Assembler assembles Fish dataset in 16 minutes, while it takes 26 minutes with 2048 cores to assemble the Yanhuang dataset. The speedup curves of SWAP-Assembler on processing five datasets are shown in Figure 11. It shows that the speedup of assembling two small datasets have a turning point at 512 cores, and linear speedup to 1024 cores is achieved for other three larger datasets. SWAP-Assembler can still benefits from the increasing cores up to 2048 cores on processing Yanhuang dataset.

Memory footprint is a bottleneck for assembling large genomes, and parallel assemblers is a solution for large genome assembly by using more memory on the computational nodes. For our case, Fish and Yanhuang genome assembly needs 1.6T bytes and 1.8T bytes memory, respectively. As in Tianhe 1A each server has 24G memory, Fish genomes cannot be assembled on a cluster with 64 servers. The same reasoning applies to Yanhuang dataset.

SWAP-Assembler has better scalability compared with Ray and ABySS due to two important improvements. Firstly, computational interdependence of edge merging operations on one single unanimous path is resolved by MSG. Secondly, SWAP computational framework can trigger parallel computation of all operations without interference, and the communication latency is hidden by improving the computing throughput. Ray and ABySS cannot merge the *k*-mers in a single linear chain in parallel, and PASHA can only parallelize the *k*-mer merging work on different chains, which limits their degree of parallelism.

### Assembly quality assessment

This part evaluate the assembly quality of SWAP-Assembler. To be compatible with the comparison results from GAGE, we follow the error correction method of GAGE. ALLPATH-LG [35] and Quake [36] are used to correct errors for *S. aureus *and *R. sphaeriodes *datasets. The corrected reads are used as the input to ABySS, Ray and SWAP-Assembler. In addition, two other sequential genome assemblers, Velvet and SOAPdenovo, are selected in this experiment for comparison, and a machine with 1TB memory is used. The *k*-mer size for all assemblers varies between 19 and 31, and best assembly results from the experiments of different *k*-mer sizes for each assembler are reported in Table 7 and Table 8.

**Table 7:** Assembly results of *S.aureus *and *R. sphaeriodes *datasets.

Software	*S. aureus*	Contigs			*R. sphaeroides *Contigs			
	**Num**	**N50(kb)**	**Errors**	**N50 corr. (kb)**	**Num**	**N50(kb)**	**Errors**	**N50 corr.(kb)**

Velvet	162	48.4	28	41.5	583	15.7	35	**14.5**
SOAPdenovo	**107**	**288.2**	48	**62.7**	**204**	**131.7**	414	14.3
ABySS	302	29.2	14	24.8	1915	5.9	55	4.2
Ray	221	36.6	15	35.6	752	11.5	17	11.2
SWAP-Assembler	183	51.1	**3**	37.8	529	16.2	**7**	12.3

**Table 8:** Contig statistics on the assembly results of *S.aureus, R. sphaeroides *datasets. (Unit: Percentage %).

Dataset	Software	Assembly	Chaff	Unalign	Unalign	Duplicate	Compress
		**Size**	**Size**	**Ref bases**	**ASM bases**	**Ref bases**	**Ref bases**

*S. aureus *	Velvet	99.2	0.45	0.79	0.03	0.1	1.28
(2.9 Mb)	SOAPdenovo	101.3	0.35	**0.38**	**0.01**	1.44	1.41
	ABySS	127	66	1.37	*<***0.01**	23.3	**0.99**
	Ray	98.4	**0.1**	0.88	0.04	**0.08**	1.26
	SWAP-Assembler	**99.3**	0.28	0.8	0.02	1.29	1.45
	Velvet	97.8	0.54	1.6	0.01	0.29	0. 92
	SOAPdenovo	**99.9**	0.45	**0.88**	0.02	1.07	0.51
*R. sphaeroides *(4.6 Mb)	ABySS	108	1.65	3.01	0.15	10.04	**0.04**
	Ray	99	**0.13**	1.03	*<***0.01**	**0.27**	0.73
	SWAP-Assembler	99.1	**0.13**	1.08	0.11	0.83	0.75

Table 7 presents the results of four metrics for evaluation: the number of contigs, N50, number of erroneous contigs and error-corrected N50. Error-corrected N50 is used to exclude the misleading assessment of larger N50 by correcting erroneously concatenated contigs. Each erroneous contig is broken at every misjoin and at every indel longer than 5 bases. From Table 7, SWAP-Assembler generates 3 and 7 error contigs on *S. aureus *and *R. sphaeriodes *datasets, respectively, which are the smallest compared with other assemblers. N50 size and error-corrected N50 size for SWAP-Assembler are also longer than two other parallel assemblers, Ray and ABySS. SOAPdenovo has minimal number of contigs and largest N50 size for both datasets.

Table 8 summarizes the statistics of contigs generated for these two datasets. Three metrics in [30] are used to evaluate the quality of the contigs. In this table, "assembly size" close to its genome size is better. Larger "chaff size" can be indicative of the assembler being unable to integrate short repeat structures into larger contigs or not properly correcting erroneous bases. Coverage can be measured by the percentage of reference bases aligned to any assembled contig, which is "100%-Unaligned ref bases" [30]. SWAP-Assembler and Ray both have the smallest chaff size of 0.13% on *R. sphaeroides *dataset, and they show very close coverage and assembly sizes. In terms of *S. aureus *dataset, Ray has a lower chaff size of 0.10% compared with SWAP-Assembler, however, SWAP-Assembler generates better assembly size of 99.3% and larger coverage of 99.2%.

We also analyzed the contig statistics for three larger datasets and the results are presented in Table 9. Because these datasets do not have a standard reference set and the original script provided by GAGE requires a reference set, we wrote a script to analyze the assembly results using the number of contigs, N50 size, max length of contigs and bases in the contigs for evaluation. The original data of three datasets are directly processed by five assemblers with a fixed *k*-mer size of 31. According to Table 9, the N50 size of contigs generated by SWAP-Assembler is longest for all three datasets. For Fish and Yanhuang datasets, SWAP-Assembler also performs best in the number of contigs and max length of contigs. However for SWAP-Assembler on Hg14 dataset, whose reads are extracted from the human dataset by mapping the human chromosome 14, the number of contigs, max length of contigs and bases in contigs have a rank of second, third, and second, respectively. SWAP-Assembler has a best N50 size for all datasets. This is because it has efficient graph cleaning and contig extension steps, which can handle sequencing errors efficiently. Four other assemblers, without the help from external tools on error correction, are affected by the quality of input reads on larger datasets.

**Table 9:** Contig statistics of Hg14, Fish and Yanhuang datasets.

Dataset	Software	Contigs			
		**Num**	**N50 (bp)**	**Max (bp)**	**BasesInContig (Mbp)**

	Velvet	90,784	1688	25,729	**83.3**
Hg 14 (88.3 Mb)	SOAPdenovo	200,153	836	21,144	96.4
	ABySS	190,693	1914	**26,697**	107.4
	Ray	**76,950**	964	14,399	68.4
	SWAP-Assembler	88,609	**2036**	21,246	96.4
Fish (1 Gb)	Velvet	-	-	-	-
	SOAPdenovo	3291290	378	7181	1,134.40
	ABySS	-	-	-	-
	Ray	-	-	-	-
	SWAP-Assembler	**2881443**	**1309**	**35,962**	**1,097.90**
	Velvet	-	-	-	-
Yanhuang (3 Gb)	SOAPdenovo	8,584,515	841	23,782	**3396.2**
	ABySS	9,218,967	1059	24,428	3691.8
	Ray	3,755,103	266	6,765	1620.1
	SWAP-Assembler	**2,379,151**	**1368**	**31,152**	2434.3

In conclusion, SWAP-Assembler is a highly scalable and efficient genome assembler. The evaluation shows that our assembler can scales up to 2048 cores, which is much better than other parallel assemblers, and the quality of contigs generated by SWAP-Assembler is the best in terms of error rate for several small datasets and N50 size for two larger data sets.

## Conclusion

In this paper, SWAP-Assembler, a fast and efficient genome assembler scaling up to thousands of cores, is presented. In SWAP-Assembler, two fundamental improvements are crucial for its scalability. Firstly, MSG is presented as a comprehensive mathematical abstraction for genome assembly. With MSG the computational interdependence is resolved. Secondly, SWAP computational framework triggers the parallel computation of all operations without interference. Two additional steps are included to improve the quality of contigs. One is graph cleaning, which adopts the traditional methods of removing *k*-molecules and edges with low frequency, and the other is contig extension, which resolves special edges and some cross nodes with a heuristic method. Results show that SWAP-Assembler can scale up to 2048 cores on Yanhuang dataset using only 26 minutes, which is the best compared to other parallel assemblers, such as ABySS and Ray. Conitg evaluation results confirm that SWAP-Assembler can generate good results on contigs N50 size and retain low error rate. When processing massive datasets without using external error correction tools, SWAP-Assembler is immune from low data quality and generated longest N50 contig size.

For large genome and metagenome data of Tara bytes, for example the human gut microbial community sequencing data, highly scalable and efficient assemblers will be essential for data analysis. Our future work will extend our algorithm development for massive matagenomics dataset with additional modules.

The program can be downloaded from https://sourceforge.net/projects/swapassembler.

## Competing interests

The authors declare that they have no competing interests.

## Authors' contributions

JT carried out the parallel genome assembly studies, participated in the development of SWAP-Assembler and drafted the manuscript. BQ participated in the design and optimization of SWAP-Assembler. YJ participated in the development of SWAP-Assembler and modification of this manuscript. SZ participated in the design of the study and design of the performance test. Pavan conceived of the study, and participated in its design and coordination and helped to draft the manuscript. All authors read and approved the final manuscript.

## Additional file 1

Appendix. Appendix 1 property proof of MSG, Appendix 2 algorithms for SWAP-Assembler, Appendix 3 complexity analysis of SWAP computational framework, Appendix 4 complexity analysis of graph reduction.

## Declarations

Funding for the publication of this article was provided by National Science Foundation of China under grant No. 11204342, the Science Technology and Innovation Committee of Shenzhen Municipality under grant No. JCYJ20120615140912201, and Shenzhen Peacock Plan under grant No. KQCX20130628112914299.

This article has been published as part of BMC Bioinformatics Volume 15 Supplement 9, 2014: Proceedings of the Fourth Annual RECOMB Satellite Workshop on Massively Parallel Sequencing (RECOMB-Seq 2014). The full contents of the supplement are available online at http://www.biomedcentral.com/bmcbioinformatics/supplements/15/S9.
